# Sexual dimorphism in cardiometabolic and cardiac mitochondrial function in obese rats following sex hormone deprivation

**DOI:** 10.1038/s41387-022-00189-0

**Published:** 2022-03-17

**Authors:** Krekwit Shinlapawittayatorn, Wanpitak Pongkan, Sivaporn Sivasinprasasn, Siriporn C. Chattipakorn, Nipon Chattipakorn

**Affiliations:** 1 Cardiac Electrophysiology Research and Training Center, Faculty of Medicine, Chiang Mai University, Chiang Mai, 50200 Thailand; 2 Cardiac Electrophysiology Unit, Department of Physiology, Faculty of Medicine, Chiang Mai University, Chiang Mai, 50200 Thailand; 3 Center of Excellence in Cardiac Electrophysiology Research, Chiang Mai University, Chiang Mai, 50200 Thailand; 4 Department of Veterinary Biosciences and Veterinary Public Health, Faculty of Veterinary Medicine, Chiang Mai University, Chiang Mai, 50200 Thailand; 5 School of Medicine, Mae Fah Luang University, Chiang Rai, 57100 Thailand; 6 Department of Oral Biology and Diagnostic Sciences, Faculty of Dentistry, Chiang Mai University, Chiang Mai, 50200 Thailand

**Keywords:** Cardiovascular diseases, Preclinical research

## Abstract

**Objective:**

Our study aims to test the hypothesis that poorer function of cardiac mitochondria in males, under sex hormone-deprived and obese-insulin-resistant conditions, is responsible for a worse cardiometabolic function than females.

**Methods:**

One hundred and forty-four rats were subjected to receive either 12 weeks of normal diet (ND) or a high-fat diet (HFD) consumption following the induction of sex hormone deprivation. Temporal evaluations of metabolic parameters, cardiac autonomic modulation, left ventricular (LV) contractile, and mitochondrial functions were measured after starting each feeding protocol for 4, 8, and 12 weeks.

**Results:**

After HFD feeding for 8 weeks, increased plasma insulin and HOMA index were initially observed in male HFD-fed sham-operated rats (M-HFS), male HFD-fed orchiectomized rats (M-HFO), female ND-fed ovariectomized rats (F-OVX), female HFD-fed sham-operated rats (F-HFS), and female HFD-fed ovariectomized rats (F-HFO) groups. In addition, as early as week 4, male ND-fed orchiectomized rats (M-ORX) and M-HFO exhibited impaired cardiac autonomic balance, LV contractile and mitochondrial functions, whereas M-HFS and F-HFO developed these impairments at week 8 and F-OVX and F-HFS exhibited them at week 12.

**Conclusion:**

We concluded that sex hormone-deprived females are prone to develop metabolic impairments, whereas males are more likely to have cardiac autonomic impairment, LV contractile and mitochondrial dysfunction even in the absence of obese-insulin-resistant condition. However, under estrogen-deprived condition, these impairments were further accelerated and aggravated by obese-insulin resistance.

## Introduction

Sex differences have been shown to influence the development of many diseases including cardiovascular diseases (CVD) [1–4]. In addition, the prevalence, clinical presentation, severity and outcome for CVD vary based on gender [2]. Although the incidence of CVD is significantly less common in women, the incidence raises after menopause suggesting that this significant sex difference is a key contributor to the CVD benefit [5]. For men, the testosterone level substantially declines [6] and the incidence of several cardiometabolic disorders including metabolic syndrome increases with age [7, 8]. In addition, our previous study demonstrated that orchiectomized (ORX) rats demonstrated impaired left ventricular (LV) contractile function and cardiac autonomic dysregulation [9]. For women, a bilateral ovariectomy (OVX) significantly increased CVD mortality, suggesting the cardioprotective actions of estrogen [10]. Moreover, estrogen deprivation could induce myocardial contractile dysfunction in female rats [11].

In addition to sex hormone deprivation, obesity has been shown to increase the risk for CVD [12]. We previously demonstrated that rats developed obese-insulin resistance [13], cardiac autonomic imbalance, LV contractile dysfunctions, increased production of reactive oxygen species (ROS), and impaired mitochondrial function [14] after feeding with a high-fat diet (HFD) for 8 weeks. Consistently, clinical evidence also demonstrates that after menopause the metabolic syndrome incidence among women increased dramatically [15]. Because sex differences in CVD are prevalent, it is therefore necessary to study the impact of sexual dimorphism on cardiometabolic and cardiac mitochondrial function. Although either sex hormone deprivation or obese-insulin resistance has been shown to affect cardiac function, the link between sexual dimorphism in cardiometabolic parameters, cardiac sympathovagal activity, LV contractile and mitochondrial functions remain poorly understood, especially the temporal effects of combined conditions.

We hypothesized that under both sex hormone-deprived and obese-insulin-resistant conditions, males have a poorer cardiometabolic function and worse cardiac mitochondrial function than females. Understanding sex-related differences in the phenotypic expression of cardiometabolic and cardiac mitochondrial function is important because it is possible that different therapies and treatments could be targeted for men and women, especially in the presence of combined conditions.

## Material and methods

### Animals

The experimental studies were conducted in accordance with the NIH guidelines for the use of laboratory animals and were approved by the Animal Care and Use Committee (Chiang Mai University). Both male and female 5–6 weeks old Wistar rats were provided by the National Laboratory Animal Center (Mahidol University, Bangkok, Thailand). Under the temperature of 22–25 °C, rats were housed one rat per cage with a light-dark (12h–12h) cycle. Rats were fed (ad libitum) with either a normal diet (ND) (Mouse Feed Food No. 082, C.P. Company, Thailand) or an HFD.

### Orchiectomy and ovariectomy procedures

Isoflurane (2–3%) was used as an anesthetic agent. Male rats were subjected to orchiectomy (ORX) as described previously [16]. Female rats were subjected to the bilateral ovariectomy (OVX) procedure as previously described [17]. Then, rats were subcutaneously injected with analgesic drugs and antibiotics for 3 days. Then, a clear box with dry bedding was used to house rats individually. Rats in the ND group were fed with standard laboratory chow (19.77% E from fat). In contrast, rats in the HFD group were fed with an HFD (59.28% E from fat). The ingredients of HFD are as follows: standard rat diet, cholesterol, casein, DL-methionine, lard, NaCl, vitamins, and yeast powder [13].

### Experimental design

One hundred and forty-four rats were randomly subjected to a sham-operated group and ORX or OVX group. Rats were returned to the cage for recovery for 1 week after the operation. Then, rats in each group (*n* = 6/group for each duration in each dietary group) were subjected to receive different dietary consumption (ND or HFD) for 4, 8, and 12 weeks. Thus, there are eight experimental groups as follow: (1) male ND-fed sham-operated rats (M-NDS), (2) male ND-fed orchiectomized rats (M-ORX), (3) male HFD-fed sham-operated rats (M-HFS), (4) male HFD-fed orchiectomized rats (M-HFO), (5) female ND-fed sham-operated rats (F-NDS), (6) female ND-fed ovariectomized rats (F-OVX), (7) female HFD-fed sham-operated rats (F-HFS), and (8) female HFD-fed ovariectomized rats (F-HFO). Both body weight and food consumption were daily noted, and blood specimens were collected at 4^th^, 8^th^, and 12^th^ weeks after 5–6 h of fasting. The assessment of food consumption was performed by using the manually weighing method. Food consumption was recorded by manually weighing a food dish before and after a feeding period. The plasma of blood specimens was used for the assessment of metabolic parameters. The ex vivo mass of visceral fat was dissected and assessed by measuring the weight of the total perirenal and peri-epididymal adipose tissues [16, 18].

### Determination of HRV

The cardiac autonomic balance was accessed by determining heart rate variability (HRV) [19]. An ECG was performed at 4^th^, 8^th^, and 12^th^ weeks in each rat after being fed with an ND or HFD. The ECG was continuously recorded for 20 min using PowerLab (PowerLab 4/25T, AD Instruments, Australia) with chart 5.0, while the animals were still conscious. MATLAB program was used to analyze the ECG data [20]. The analysis of HRV was presented in the frequency domain as follows: A high frequency (HF, 0.6–0.3 Hz) and a low frequency (LF, 0.2–0.6 Hz), which indicates vagal and sympathovagal activities, respectively [19]. The balance of cardiac sympathovagal activity was evaluated by using the LF/HF ratio [21]. The autonomic shift toward sympathetic activity was identified by an increase in LF/HF ratio [14].

### Determination of cardiac function

Rats were anesthetized by isoflurane inhalation (2% isoflurane, 98% O_2_). Then, rats were restrained by fixing the four limbs to a small animal surgery board using a fixing tape. An echocardiograph (SONOS4500, Philips) was used to determine LV function. The LV function was analyzed at baseline and after feeding protocol. The LV ejection fraction (EF) was measured by using standard M-mode echocardiography.

### Surrogate measures of cardiac mitochondrial function

Both 50 mg/kg of zoletil and 3 mg/kg of xylazine were used as an anesthetic. The isolated cardiac mitochondria were collected and the protein concentration was measured as described previously [22]. After the application of 2 mM of H_2_O_2_, 2 μM of dichlorohydrofluorescein diacetate dye was used to determine the percent change in cardiac mitochondrial ROS production [22]. A fluorescent microplate reader (BioTek Instruments, Winooski, USA) was used to determine the ROS level at the excitation wavelength (λ_ex_) and the emission wavelength (λ_em_) at 485 nm (bandwidth 5 nm) and 530 nm (bandwidth 10 nm), respectively. Increased cardiac mitochondrial ROS generation is indicated by increased fluorescent intensity [23].

After application of 2 mM of H_2_O_2_, the percent change of cardiac mitochondria membrane potential were determined by staining with 5 μM of mitochondrial probe 5,5′,6,6′ tetrachloro-1,1′,3,3′-tetraethylbenzimidazolcarbocyanine iodide (JC-1) [22] and determined by a microplate reader for fluorescent detection. The depolarization of cardiac mitochondria was identified as a decreased red/green fluorescent ratio [23].

The level of mitochondrial swelling was determined from a suspension of isolated cardiac mitochondria using a microplate reader (Synergy HT, BioTek). Mitochondria (0.4 mg/mL) were incubated in respiration buffer as previously described [22]. The swelling of cardiac mitochondria was determined by a decrease in the absorbance by the mitochondrial suspension at 540 nm [23].

### Determination of metabolic parameters, insulin resistance, sex hormone, and plasma and cardiac malondialdehyde levels

Metabolic parameters were measured by an enzymatic colorimetric assay using a commercially available kit (Biotech, Bangkok, Thailand). Fasting plasma HDL, LDL, and insulin levels were measured using a sensitive, colorimetric and fluorometric assay (BioVision), Friedewald’s equation [24] and Sandwich ELISA (Millipore), respectively.

Insulin resistance was determined using the homeostasis model assessment (HOMA) [25]. A higher HOMA index indicates a greater degree of insulin resistance [13]. Total plasma testosterone and estradiol levels were determined by ELISA and a competitive enzyme immunoassay kit (Cayman Chemical Company, MI, USA), respectively.

Plasma and cardiac malondialdehyde (MDA) levels were determined using an HPLC system (Thermo Scientific) [18]. The concentrations of either plasma or cardiac thiobarbituric acid reactive substances (TBARS) were obtained from a standard curve, and reported as an MDA equivalent concentration [26].

### Statistical analysis

All values were presented as mean ± SEM. For cardiac function, LF/HF ratio and the surrogate measures of mitochondrial function. SPSS program (SPSS version 23, SPSS Inc.) was used to determine the significance of the difference between the mean by using a three-way-ANOVA for each time period (4, 8, and 12 weeks) with gender, hormone, and diet as the three factors, and was followed by pairwise Tukey’s tests. The difference between the mean of the blood serum testosterone and estradiol levels were determined using the unpaired Student’s *t*-test. The difference between the groups for the rest of the parameters was analyzed by two-way ANOVA, followed by LSD post-hoc analysis. *P* < 0.05 was used as a criterion of statistical significance.

## Results

### Sex-related differences in the development of insulin resistance in the presence of sex hormone deprivation

At week 4, sex hormone deprivation was confirmed by decreased plasma testosterone (Table 1) or estradiol (Table 2) levels. Orchiectomized or ovariectomized rats in both dietary groups (M-ORX, M-HFO, F-OVX, and F-HFO) had significantly decreased plasma testosterone or estradiol levels, when compared with sex-matched NDS at the same time points. The body weight of M-HFS and F-HFS were significantly increased compared with sex-matched NDS. In contrast, the body weight, food intake, and visceral fat of M-ORX, but not F-OVX, were decreased when compared with M-NDS. However, the metabolic parameters were not different between M-ORX, M-HFS, M-HFO, F-OVX, F-HFS, and F-HFO compared with sex-matched NDS (Tables 3–5).

**Table 1:** Summary of blood serum testosterone levels.

Experimental groups		Week		
		4	8	12
Blood serum testosterone (ng/dL)	M-NDS	1.37 ± 0.68	1.61 ± 0.83	1.10 ± 0.16
	M-ORX	0.07 ± 0.12*^,†^	0.04 ± 0.01*^,†^	0.06 ± 0.01*^,†^
	M-HFS	0.99 ± 0.41^‡^	1.05 ± 0.27^‡^	0.50 ± 0.06*^,‡^
	M-HFO	0.11 ± 0.01*^,†^	0.11 ± 0.01*^,†^	0.08 ± 0.01*^,†^

*M-NDS* male normal diet sham, *M-ORX* male orchiectomy, *M-HFS* male high-fat diet sham, *M-HFO* male high-fat diet with orchiectomy.

*P < 0.05 vs. age-matched M-NDS, ^†^*P* < 0.05 vs. age-matched M-HFS, ^‡^*P* < 0.05 vs. age-matched M-ORX.

**Table 2:** Summary of blood serum estradiol levels.

Experimental groups		Week		
		4	8	12
Blood serum estradiol (ng/dL)	F-NDS	13.9 ± 3.48	11.3 ± 1.43	13.1 ± 1.72
	F-OVX	4.33 ± 1.06*^,†^	5.16 ± 0.31*^,†^	6.39 ± 0.79*^,†^
	F-HFS	13.76 ± 2.03^‡^	10.26 ± 0.83^‡^	13.5 ± 1.19^‡^
	F-HFO	3.06 ± 0.24*^,†^	5.67 ± 0.49*^,†^	6.25 ± 0.46*^,†^

*F-NDS* female normal diet sham, *F-OVX* female ovariectomy, *F-HFS* female high-fat diet sham, *F-HFO* female high-fat diet with ovariectomy.

*P < 0.05 vs. age-matched F-NDS, ^†^*P* < 0.05 vs. age-matched F-HFS, ^‡^*P* < 0.05 vs. age-matched F-OVX.

**Table 3:** Effects of sex hormone on metabolic parameters in normal diet-fed male and female rats.

Metabolic parameters	4 weeks of dietary consumption				8 weeks of dietary consumption				12 weeks of dietary consumption			
	Male		Female		Male		Female		Male		Female	
	M-NDS	M-ORX	F-NDS	F-OVX	M-NDS	M-ORX	F-NDS	F-OVX	M-NDS	M-ORX	F-NDS	F-OVX
Body weight (g)	444 ± 14.3	420 ± 9.0*	270 ± 10.5	281 ± 6.0	452 ± 6.6	443 ± 8.6*	278 ± 5.8	305 ± 5.9*	485 ± 8.6	424 ± 6.8*	286 ± 4.0	334 ± 2.9*
Food intake (g/day)	23 ± 0.6	18 ± 0.4*	16 ± 0.5	16 ± 1.1	22 ± 0.5	16 ± 0.7*	15 ± 1.3	14 ± 1.1	21 ± 0.6	17 ± 0.9*	12 ± 0.7	14 ± 1.3
Visceral fats (g)	24 ± 1.5	17 ± 2.2*	12 ± 1.0	10 ± 1.0	30 ± 1.0	16 ± 0.6*	10 ± 0.5	10 ± 1.0	28 ± 1.9	22 ± 0.6*	11 ± 0.3	15 ± 0.9*
Plasma glucose (mg/dL)	126 ± 3.1	128 ± 8.2	123 ± 10.9	124 ± 10.9	125 ± 7.9	135 ± 8.2	127 ± 6.0	127 ± 6.0	136 ± 3.8	135 ± 5.1	124 ± 5.8	118 ± 6.9
Plasma insulin (ng/mL)	2.1 ± 0.4	2.0 ± 0.3	0.8 ± 0.1	0.9 ± 0.1	1.9 ± 0.2	1.9 ± 0.1	1.1 ± 0.1	1.6 ± 0.2*	2.1 ± 0.2	2.1 ± 0.2	0.8 ± 0.1	1.5 ± 0.3*
HOMA index	12.1 ± 2.1	10.7 ± 1.2	5.7 ± 0.4	6.1 ± 0.8	10.7 ± 1.4	11.6 ± 0.9	5.9 ± 1.0	10.7 ± 1.5*	13.4 ± 1.4	12.4 ± 1.0	5.2 ± 0.4	12.4 ± 2.3*
Cholesterol (mg/dL)	89 ± 3.5	91 ± 5.0	99 ± 6.7	98 ± 8.5	91 ± 4.1	93 ± 4.3	109 ± 6.1	106 ± 2.8	89 ± 4.7	100 ± 3.2	92 ± 4.6	115 ± 15.1
HDL (mg/dL)	30 ± 1.0	32 ± 1.6	31 ± 1.3	33 ± 1.9	34 ± 1.8	33 ± 0.3	31 ± 1.9	34 ± 0.6	33 ± 0.3	34 ± 1.6	29 ± 1.2	29 ± 1.5
LDL (mg/dL)	60 ± 2.6	65 ± 1.5	76 ± 5.3	78 ± 6.7	58 ± 4.5	68 ± 6.6	78 ± 5.1	89 ± 3.4	63 ± 4.6	73 ± 7.5	71 ± 8.0	79 ± 3.9
Triglyceride (mg/dL)	135 ± 12.1	139 ± 8.1	35 ± 3.4	30 ± 2.4	129 ± 7.7	130 ± 8.4	45 ± 4.0	41 ± 3.0	133 ± 12.1	135 ± 10.2	40 ± 4.1	41 ± 3.4
Serum MDA (µmol/mL)	4.6 ± 0.03	5.3 ± 0.12*	3.5 ± 0.42	3.6 ± 0.37	5.0 ± 0.07	5.7 ± 0.12*	3.5 ± 0.31	3.8 ± 0.55	4.2 ± 0.4	5.2 ± 0.05*	3.5 ± 0.21	4.1 ± 0.28*
Cardiac MDA (µmol/mL)	1.5 ± 0.26	3.1 ± 0.76*	2.4 ± 0.43	2.6 ± 0.61	2.0 ± 0.22	4.7 ± 1.01*	2.6 ± 0.39	2.5 ± 0.20	2.3 ± 0.60	6.1 ± 0.75*	4.3 ± 0.81	6.3 ± 0.76*

*HDL* high-density lipoprotein, *HOMA* homeostatic model assessment, *LDL* low-density lipoprotein, *M-NDS* male normal diet sham, *MDA* malondialdehyde, *M-ORX* male orchiectomy, *F-NDS* female normal diet sham, *F-OVX* female ovariectomy.

*P < 0.05 vs. age-matched NDS in the same gender.

**Table 4:** Effects of dietary consumption on metabolic parameters in male and female rats.

Metabolic parameters	4 weeks of dietary consumption				8 weeks of dietary consumption				12 weeks of dietary consumption			
	Male		Female		Male		Female		Male		Female	
	M-NDS	M-HFS	F-NDS	F-HFS	M-NDS	M-HFS	F-NDS	F-HFS	M-NDS	M-HFS	F-NDS	F-HFS
Body weight (g)	444 ± 14.3	482 ± 12.4*	270 ± 10.5	285 ± 7.7*	452 ± 6.6	526 ± 14.7*	278 ± 5.8	306 ± 10.3*	485 ± 8.6	588 ± 19.8*	286 ± 4.0	344 ± 8.7*
Food intake (g/day)	23 ± 0.6	24 ± 0.1	16 ± 0.5	17 ± 0.4	22 ± 0.5	23 ± 0.1	15 ± 1.3	16 ± 0.6	21 ± 0.6	22 ± 0.5	12 ± 0.7	16 ± 0.8*
Visceral fats (g)	24 ± 1.5	35 ± 2.2*	12 ± 1.0	20 ± 1.6*	30 ± 1.0	51 ± 2.5*	10 ± 0.5	25 ± 1.6*	28 ± 1.9	51 ± 4.6*	11 ± 0.3	32 ± 2.0*
Plasma glucose (mg/dL)	126 ± 3.1	130 ± 8.2	123 ± 10.9	125 ± 12.5	125 ± 7.9	127 ± 5.2	127 ± 6.0	134 ± 8.3	136 ± 3.8	133 ± 4.4	124 ± 5.8	135 ± 10.0
Plasma insulin (ng/mL)	2.1 ± 0.4	2.1 ± 0.2	0.8 ± 0.1	0.9 ± 0.1	1.9 ± 0.2	2.9 ± 0.1*	1.1 ± 0.1	1.5 ± 0.2*	2.1 ± 0.2	3.4 ± 0.1*	0.8 ± 0.1	1.5 ± 0.2*
HOMA index	12.1 ± 2.1	11.2 ± 0.9	5.7 ± 0.4	6.5 ± 1.7	10.7 ± 1.4	16.6 ± 1.0*	5.9 ± 1.0	13.9 ± 3.4*	13.4 ± 1.4	20.1 ± 1.0*	5.2 ± 0.4	13.3 ± 2.9*
Cholesterol (mg/dL)	89 ± 3.5	96 ± 4.1	99 ± 6.7	99 ± 10.6	91 ± 4.1	104 ± 5.5*	109 ± 6.1	101 ± 3.5	89 ± 4.7	109 ± 5.8*	92 ± 4.6	134 ± 13.3*
HDL (mg/dL)	30 ± 1.0	29 ± 1.7	31 ± 1.3	28 ± 2.1	34 ± 1.8	27 ± 1.6*	31 ± 1.9	29 ± 1.8	33 ± 0.3	29 ± 2.1*	29 ± 1.2	28 ± 1.4
LDL (mg/dL)	60 ± 2.6	67 ± 5.3	76 ± 5.3	74 ± 8.6	58 ± 4.5	77 ± 8.1*	78 ± 5.1	83 ± 4.6	63 ± 4.6	82 ± 7.6*	71 ± 8.0	85 ± 15.2
Triglyceride (mg/dL)	135 ± 12.1	138 ± 15.6	35 ± 3.4	36 ± 4.0	129 ± 7.7	116 ± 17.7	45 ± 4.0	46 ± 1.4	133 ± 12.1	146 ± 8.8	40 ± 4.1	42 ± 3.1
Serum MDA (µmol/mL)	4.6 ± 0.03	5.0 ± 0.1	3.5 ± 0.42	3.5 ± 0.09	5.0 ± 0.07	5.8 ± 0.14*	3.5 ± 0.31	3.7 ± 0.48	4.2 ± 0.4	5.9 ± 0.78*	3.5 ± 0.21	5.9 ± 0.78*
Cardiac MDA (µmol/mL)	1.5 ± 0.26	1.5 ± 0.15	2.4 ± 0.43	2.3 ± 0.04	2.0 ± 0.22	4.4 ± 0.05*	2.6 ± 0.39	2.8 ± 0.81	2.3 ± 0.60	5.6 ± 0.24*	4.3 ± 0.81	6.4 ± 0.26*

*HDL* high-density lipoprotein, *HOMA* homeostatic model assessment, *LDL* low-density lipoprotein, *MDA* malondialdehyde, *M-NDS* male normal diet sham, *M-HFS* male high-fat diet sham, *F-NDS* female normal diet sham, *F-HFS* female high-fat diet sham.

*P < 0.05 vs. age-matched NDS in the same gender.

**Table 5:** Effects of dietary consumption combined with sex hormone deprivation on metabolic parameters in male and female rats.

Metabolic parameters	4 weeks of dietary consumption				8 weeks of dietary consumption				12 weeks of dietary consumption			
	Male		Female		Male		Female		Male		Female	
	M-NDS	M-HFO	F-NDS	F-HFO	M-NDS	M-HFO	F-NDS	F-HFO	M-NDS	M-HFO	F-NDS	F-HFO
Body weight (g)	444 ± 14.3	458 ± 12.0	270 ± 10.5	327 ± 4.4*	452 ± 6.6	498 ± 11.6*	278 ± 5.8	388 ± 8.5*	485 ± 8.6	508 ± 19.6	286 ± 4.0	422 ± 9.0*
Food intake (g/day)	23 ± 0.6	23 ± 0.3	16 ± 0.5	18 ± 0.3	22 ± 0.5	21 ± 0.4	15 ± 1.3	17 ± 0.5	21 ± 0.6	20 ± 0.4	12 ± 0.7	15 ± 0.9
Visceral fats (g)	24 ± 1.5	29 ± 0.6*	12 ± 1.0	22 ± 2.2*	30 ± 1.0	37 ± 2.3*	10.5 ± 1.1	30 ± 2.2*	28 ± 1.9	28 ± 3.5	11 ± 0.3	34 ± 1.0*
Plasma glucose (mg/dL)	126 ± 3.1	126 ± 8.5	123 ± 10.9	123 ± 8.8	125 ± 7.9	135 ± 9.9	127 ± 6.0	153 ± 15.4*	136 ± 3.8	136 ± 7.3	124 ± 5.8	165 ± 6.5*
Plasma insulin (ng/mL)	2.1 ± 0.4	2.4 ± 0.4	0.8 ± 0.1	1.1 ± 0.1	1.9 ± 0.2	3.4 ± 0.7*	1.1 ± 0.1	1.7 ± 0.2*	2.1 ± 0.2	3.6 ± 0.7*	0.8 ± 0.1	1.4 ± 0.2*
HOMA index	12.1 ± 2.1	13.5 ± 1.6	5.7 ± 0.4	7.1 ± 1.4	10.7 ± 1.4	18.1 ± 3.0*,	5.9 ± 1.0	16.1 ± 1.5*	13.4 ± 1.4	20.5 ± 2.8*	5.2 ± 0.4	16.2 ± 1.7*
Cholesterol (mg/dL)	89 ± 3.5	97 ± 6.8	99 ± 6.7	105.8 ± 13.4	91 ± 4.1	114 ± 5.0*	109 ± 6.1	139 ± 3.3*	89 ± 4.7	129 ± 9.8*	92 ± 4.6	160 ± 16.1*
HDL (mg/dL)	30 ± 1.0	28 ± 2.2	31 ± 1.3	29 ± 1.3	34 ± 1.8	27 ± 3.2*	31 ± 1.9	30 ± 1.8	33 ± 0.3	30 ± 1.5*	29 ± 1.2	29 ± 1.8
LDL (mg/dL)	60 ± 2.6	65 ± 8.9	76 ± 5.3	80 ± 8.3	58 ± 4.5	90 ± 6.7*	78 ± 5.1	119 ± 3.1*	63 ± 4.6	95 ± 8.1*	71 ± 8.0	127 ± 15.7*
Triglyceride (mg/dL)	135 ± 12.1	140 ± 8.9	35 ± 3.4	32 ± 4.2	129 ± 7.7	132 ± 2.4	45 ± 4.0	46 ± 4.4	133 ± 12.1	133 ± 4.3	40 ± 4.1	42 ± 3.1
Serum MDA (µmol/mL)	4.6 ± 0.03	5.4 ± 0.26*	3.5 ± 0.42	3.3 ± 0.18	5.0 ± 0.07	5.6 ± 0.12*	3.5 ± 0.31	4.7 ± 0.16*	4.2 ± 0.4	5.4 ± 0.15*	3.5 ± 0.21	4.6 ± 0.28*
Cardiac MDA (µmol/mL)	1.5 ± 0.26	5.2 ± 0.78*	2.4 ± 0.43	3.7 ± 1.3	2.0 ± 0.22	5.1 ± 0.35*	2.6 ± 0.39	3.5 ± 0.95*	2.3 ± 0.60	7.0 ± 0.67*	4.3 ± 0.81	6.8 ± 0.76*

*HDL* high-density lipoprotein, *HOMA* homeostatic model assessment, *LDL* low-density lipoprotein, *MDA* malondialdehyde, *M-NDS* male normal diet sham, *M-HFO* male high-fat diet with orchiectomy, *F-NDS* female normal diet sham, *F-HFO* female high-fat diet with ovariectomy.

*P < 0.05 vs. age-matched NDS in the same gender.

At week 8, the body weight, food consumption, and visceral fat mass of M-ORX were not different compared with the results at week 4. In contrast, F-OVX had significantly increased body weight compared with F-NDS. Although M-ORX did not develop peripheral insulin resistance, peripheral insulin resistance was detected in F-OVX and the HFD groups (M-HFS, M-HFO, F-HFS, and F-HFO), as demonstrated by increased levels of insulin and HOMA index (Tables 3–5).

At week 12, the body weight and food consumption of M-ORX and F-OVX were similar to the results at week 8. However, the visceral fat of F-OVX was significantly increased compared with the age-matched F-NDS. M-ORX did not develop peripheral insulin resistance at week 12. In contrast, F-OVX- and HFD-treated rats (M-HFS, M-HFO, F-HFS, and F-HFO) still demonstrated consistent impairments in metabolic parameters compared with sex-matched NDS rats (Tables 3–5). In addition, the body weight of M-HFS was markedly increased compared with M-NDS, whereas the body weight and visceral fat mass were not changed or even decreased in the orchiectomized rats (M-ORX and M-HFO) compared with the age-matched M-NDS (Tables 3–5).

### Sex-related differences in the development of oxidative stress in the presence of sex hormone deprivation

At week 4, both serum and cardiac MDA were significantly increased in M-ORX and M-HFO groups compared with the age-matched NDS (Tables 3 and 5). Moreover, at week 8, both serum and cardiac MDA were significantly increased in M-ORX, M-HFS, M-HFO, and F-HFO groups compared with the age-matched NDS (Tables 3–5). At week 12, both serum and cardiac MDA were markedly increased in M-ORX, M-HFS, M-HFO, F-OVX, F-HFS, and F-HFO compared with the age-matched NDS (Tables 3–5).

### Sex-related differences in the development of cardiac dysfunction and HRV impairment in the presence of sex hormone deprivation

**Fig. 1:**
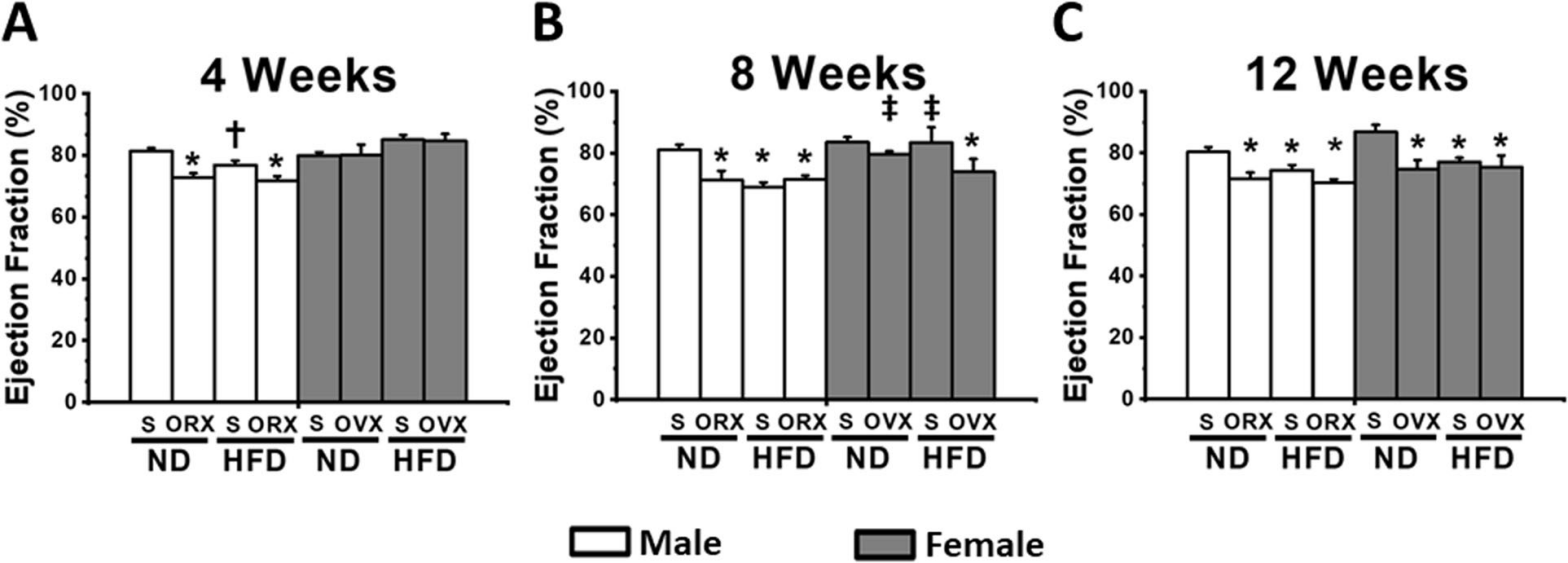
The effect of sex hormone and dietary consumption on LV function at different time courses (4, 8, and 12 weeks). **A** The LV function at 4 weeks. **B** The LV function at 8 weeks. **C** The LV function at 12 weeks. *P < 0.05 vs sex-matched ND-Sham (*n* = 6/group). ^†^*P* < 0.05 vs male orchiectomized rats (*n* = 6/group). ^‡^*P* < 0.05 vs female high-fat diet-fed ovariectomized rats (*n* = 6/group). S sham, ND normal diet, HFD high-fat diet, ORX orchiectomized rats, OVX ovariectomized rats.

For the LV contractile function of male rats, a marked decrease of %EF at week 4 was detected and continued to decrease by weeks 8 and 12 in both M-ORX and M-HFO groups, when compared with age-matched NDS (Fig. 1A–C). Unlike M-ORX and M-HFO groups, when compared with age-matched M-NDS, M-HFS demonstrated a marked reduction in %EF later at weeks 8 and 12 of the study (Fig. 1B, C).

For the LV function of female rats, the %EF was not significantly different between F-OVX and age-matched F-NDS at weeks 4 and 8 (Fig. 1A, B). However, beginning at week 8, F-HFO group exhibited a significant reduction in %EF (Fig. 1B). In addition, reduced %EF in F-OVX and F-HFS groups was not detected until week 12 (Fig. 1C). In addition, there was no statistically significant three-way interaction between gender, food and hormone at week 4 on the LV function, *F*(1, 16) = 4.268, *P* = 0.06. However, there was a statistically significant three-way interaction between gender, food and hormone at week 8 (*F*(1, 16) = 18.626, *P* = 0.001) and week 12 (*F*(1, 16) = 18.445, *P* = 0.001).

**Fig. 2:**
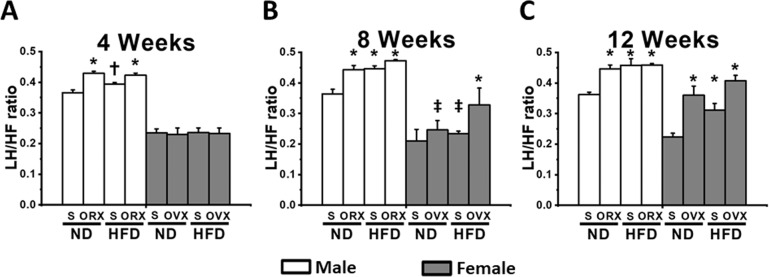
The effect of sex hormone and dietary consumption on LF/HF ratio at different time courses (4, 8, and 12 weeks). **A** The LF/HF ratio at 4 weeks. **B** The LF/HF ratio at 8 weeks. **C** The LF/HF ratio at 12 weeks. *P < 0.05 vs sex-matched ND-Sham (*n* = 6/group). *P < 0.05 vs sex-matched ND-Sham (*n* = 6/group). ^†^*P* < 0.05 vs male orchiectomized rats (*n* = 6/group). ^‡^*P* < 0.05 vs female high-fat diet-fed ovariectomized rats (*n* = 6/group). S sham, ND normal diet, HFD high-fat diet, ORX orchiectomized rats, OVX ovariectomized rats.

Consistent with the %EF profiles, an impaired cardiac autonomic balance was first detected in both M-ORX and M-HFO groups (Fig. 2A). Moreover, the ratio of LF/HF in both M-ORX and M-HFO groups markedly increased from week 4 onward when compared with the age-matched M-NDS group. Furthermore, by weeks 8 and 12, the M-HFS group exhibited cardiac autonomic dysregulation (Fig. 2B, C).

In female rats, at week 4, the cardiac autonomic regulation did not differ among groups (Fig. 2A). However, at week 8, depressed HRV was detected only in F-HFO group (Fig. 2B), suggesting that the impaired cardiac autonomic regulation was firstly developed in the F-HFO group. Moreover, when compared with the age-matched F-NDS group, increased LF/HF ratio in F-OVX, F-HFS and F-HFO groups were detected at week 12 (Fig. 2). Interestingly, a further increase in LF/HF ratio in F-HFO rats was detected at week 12 (Fig. 2C). However, a statistically significant three-way interaction between gender, food, and hormone was detected only at week 8 on the LF/HF ratio, *F*(1, 16) = 6.129, *P* = 0.025.

### Sex-related differences in the development of cardiac mitochondrial impairment in the presence of sex hormone deprivation

**Fig. 3:**
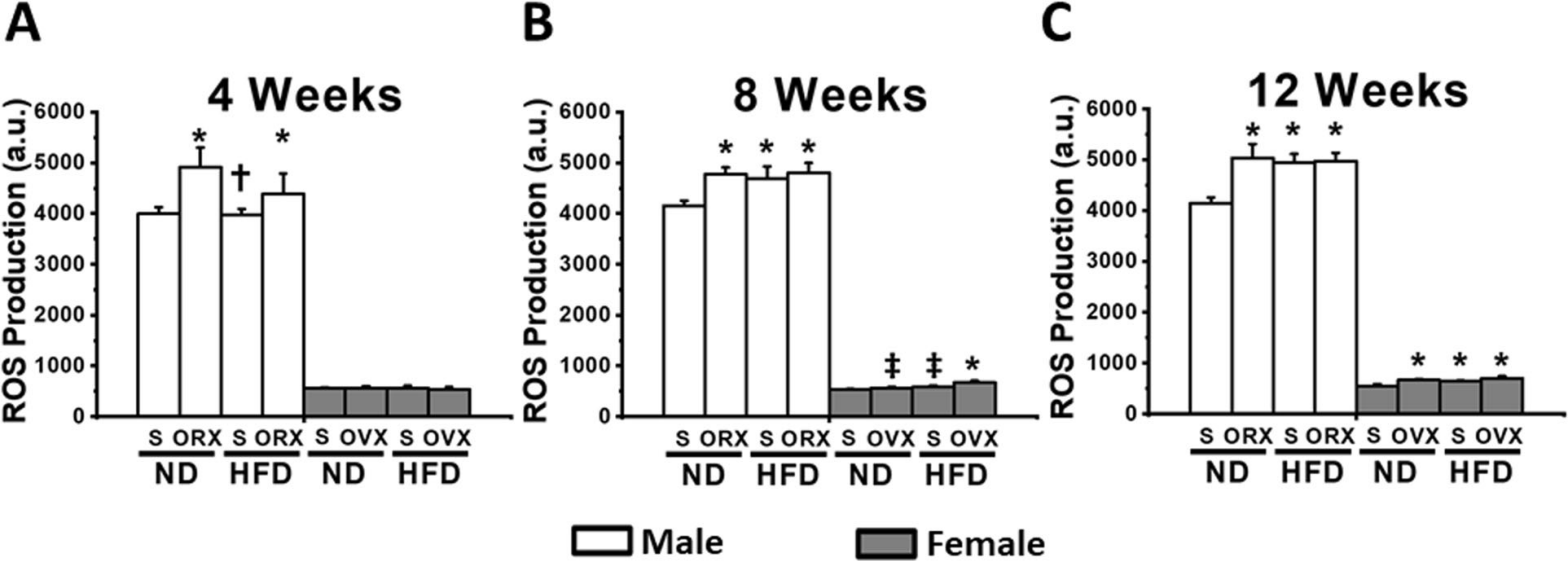
The effect of sex hormone and dietary consumption on cardiac mitochondrial ROS level at different time courses (4, 8, and 12 weeks). **A** The cardiac mitochondrial ROS level at 4 weeks. **B** The cardiac mitochondrial ROS level at 8 weeks. **C** The cardiac mitochondrial ROS level at 12 weeks. *P < 0.05 vs sex-matched ND-Sham (*n* = 6/group). *P < 0.05 vs sex-matched ND-Sham (*n* = 6/group). ^†^*P* < 0.05 vs male orchiectomized rats (*n* = 6/group). ^‡^*P* < 0.05 vs female high-fat diet-fed ovariectomized rats (*n* = 6/group). S sham, ND normal diet, HFD high-fat diet, ORX orchiectomized rats, OVX ovariectomized rats.

**Fig. 4:**
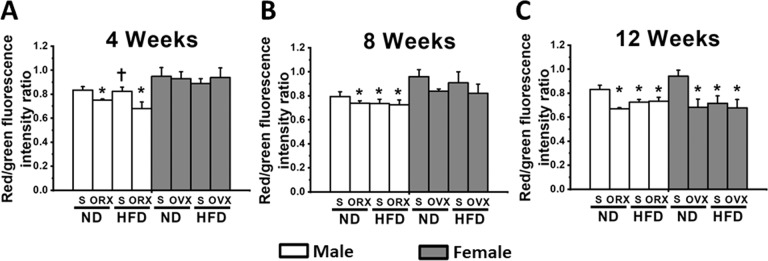
The effect of sex hormone and dietary consumption on cardiac mitochondrial membrane depolarization at different time courses (4, 8, and 12 weeks). **A** The cardiac mitochondrial membrane depolarization at 4 weeks. **B** The cardiac mitochondrial membrane depolarization at 8 weeks. **C** The cardiac mitochondrial membrane depolarization at 12 weeks. *P < 0.05 vs sex-matched ND-Sham (*n* = 6/group). ^†^*P* < 0.05 vs male orchiectomized rats (*n* = 6/group). S sham, ND normal diet, HFD high-fat diet, ORX orchiectomized rats, OVX ovariectomized rats.

**Fig. 5:**
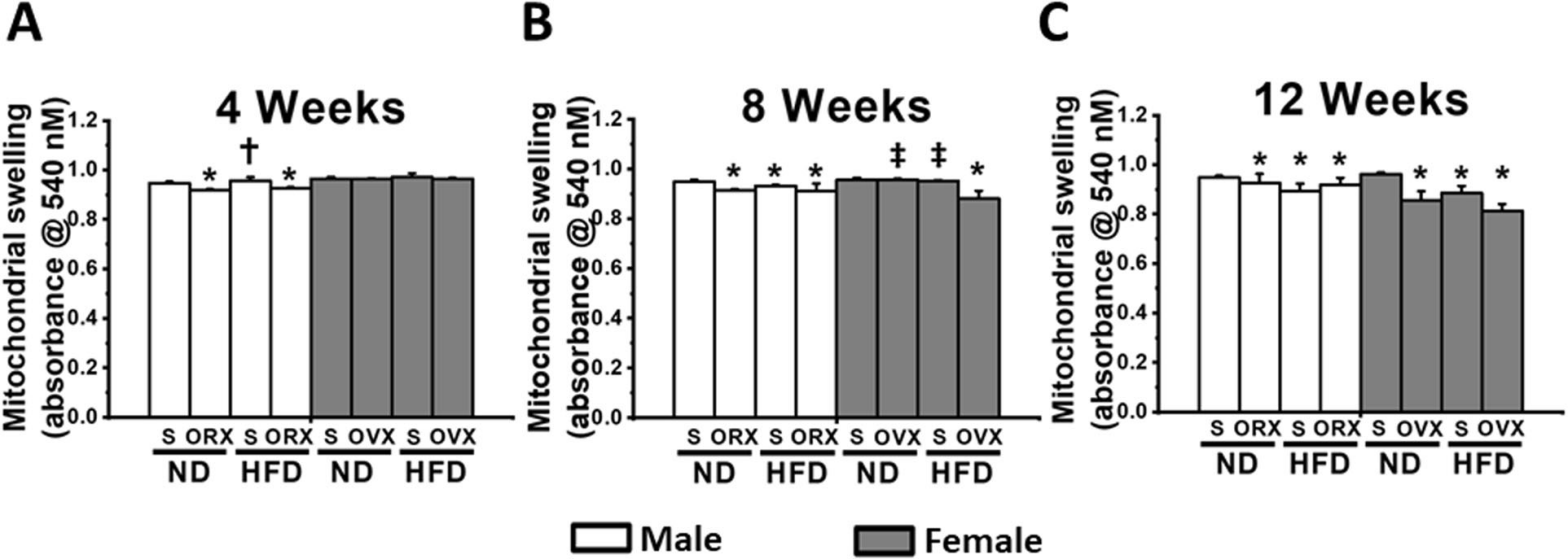
The effect of sex hormone and dietary consumption on cardiac mitochondrial swelling at different time courses (4, 8, and 12 weeks). **A** The cardiac mitochondrial swelling at 4 weeks. **B** The cardiac mitochondrial swelling at 8 weeks. **C** The cardiac mitochondrial swelling at 12 weeks. *P < 0.05 vs sex-matched ND-Sham (*n* = 6/group). *P < 0.05 vs sex-matched ND-Sham (*n* = 6/group). ^†^*P* < 0.05 vs male orchiectomized rats (*n* = 6/group). ^‡^*P* < 0.05 vs female high-fat diet-fed ovariectomized rats (*n* = 6/group). S sham, ND normal diet, HFD high-fat diet, ORX orchiectomized rats, OVX ovariectomized rats.

In male rats under sex hormone deprivation (M-ORX and M-HFO), an impaired cardiac mitochondrial function was observed early at week 4 and continued to weeks 8 and 12, as demonstrated by increased production of mitochondrial ROS level (Fig. 3), mitochondrial depolarization (Fig. 4), and mitochondrial swelling (Fig. 5) when compared with age-matched M-NDS. In M-HFS group, the cardiac mitochondrial dysfunction was detected at week 8 (Figs. 3B, 4B, and 5B).

In female rats, the surrogate of cardiac mitochondrial function did not differ among groups at week 4 (Figs. 3A, 4A, and 5A). At week 8, an impaired cardiac mitochondrial function was found only in F-HFO group (Figs. 3B, 4B, and 5B). Interestingly, both F-OVX and F-HFS rats exhibited cardiac mitochondrial dysfunction at week 12 (Figs. 3C, 4C, and 5C). In addition, for the cardiac mitochondrial ROS production, we found that there was no statistically significant three-way interaction between gender, food and hormone at week 4, *F*(1, 16) = 2.027, *P* = 0.174. However, there was a statistically significant three-way interaction between gender, food and hormone at week 8 (*F*(1, 16) = 7.742, *P* = 0.013) and week 12 (*F*(1, 16) = 12.981, *P* = 0.002). Interestingly, there was no statistically significant three-way interaction between gender, food and hormone at all time points on the alteration of cardiac mitochondrial membrane potential. For the cardiac mitochondrial swelling, there was a statistically significant three-way interaction between gender, food and hormone only at week 8, *F*(1, 16) = 10.957, *P* = 0.004.

## Discussion

The present study demonstrated that following sex hormone deprivation, there is an obvious sex-associated impact on cardiac contractile and mitochondrial functions in obese-insulin-resistant condition. Moreover, estrogen deprivation alone can cause peripheral insulin resistance in female rats, whereas peripheral insulin resistance did not observe in testosterone deprivation condition in male rats. Secondly, under sex hormone-deprived condition, females are generally more susceptible to develop metabolic disorders, whereas males are more susceptible to develop LV and cardiac mitochondrial impairments even in the absence of obese-insulin-resistant condition. Thirdly, in females, obese-insulin resistance further accelerated and aggravated these impairments under the estrogen-deprived condition. Thus, our study clearly demonstrated the sex-associated impact on cardiac contractile and mitochondrial function in obese-insulin-resistant females following estrogen withdrawal. These findings are summarized in Table 6.

**Table 6:** Summary of cardiometabolic impairment in male and female rats at different points in the feeding protocol.

Gender and experimental groups		Cardiometabolic impairment														
		Metabolic disturbance			LV dysfunction			Cardiac autonomic imbalance			Oxidative stress			Cardiac mitochondrial impairment		
		Week			Week			Week			Week			Week		
		4	8	12	4	8	12	4	8	12	4	8	12	4	8	12
Male	M-NDS															
	M-ORX				√	√	√	√	√	√	√	√	√	√	√	√
	M-HFS		√	√		√	√		√	√		√	√		√	√
	M-HFO		√	√	√	√	√	√	√	√	√	√	√	√	√	√
Female	F-NDS															
	F-OVX		√	√			√			√			√			√
	F-HFS		√	√			√			√			√			√
	F-HFO		√√	√√		√	√		√	√		√	√√		√	√

*M-NDS* male normal diet sham, *M-ORX* male orchiectomy, *M-HFS* male high-fat diet sham, *M-HFO* male high-fat diet with orchiectomy, *F-NDS* female normal diet sham, *F-OVX* female ovariectomy, *F-HFS* female high-fat diet sham, *F-HFO* female high-fat diet with ovariectomy.

### Under the deprivation of sex hormone condition, females are more prone to develop metabolic impairments than males

We found that estrogen deprivation could induce metabolic parameters impairment as early as week 8. In contrast, testosterone deprivation alone did not impair metabolic parameters, except for weight loss, at all measured time points, suggesting that females are more susceptible to develop metabolic disorders than males under sex hormone deprivation condition. Previous studies reported that girls are more insulin resistant than boys in puberty and adolescence [27, 28]. Furthermore, both Mauritius study [29] and in the RIAD cohort [30] have shown that women had more pronounced impaired glucose tolerance and lower fasting glucose levels than men. In addition, in an elderly population, the Rancho Bernardo Study has demonstrated that hyperglycemia was more frequent in women than in men [31]. The more susceptibility to develop insulin resistance in females might be explained by the more favorable fat distribution in women [32]. In our study, F-OVX, but not M-ORX had significantly increased body weight and visceral fat compared with F-NDS. A previous study has shown that increased adipocyte deposition in obesity caused an increased adipocytokine and decreased adiponectin levels [33]. Moreover, adiponectin levels are significantly reduced in obese subjects [34] and patients with type 2 diabetes [35]. Thus, it is reasonable to speculate that increased adipocytokine and decreased adiponectin levels after estrogen deprivation might be an underlying cause of peripheral insulin resistance in females.

### Males are more susceptible to develop LV and cardiac autonomic imbalance than females even in the absence of obese-insulin-resistant condition

Although testosterone deprivation in M-ORX rats did not alter metabolic parameters, it impaired LV contractile function and cardiac autonomic balance as early as week 4. In contrast, LV contractile dysfunction and cardiac autonomic imbalance in F-OVX rats was not detected until week 12, suggesting the gender-specific pathological differences in the cardiometabolic disorders. Moreover, testosterone deprivation accelerated the impairment of LV contractile function and cardiac autonomic balance in M-HFO rats. Consistently, we previously reported that testosterone deprivation accelerates, but not aggravates, the impairment of LV function and cardiac autonomic regulation in obese-orchiectomized male rats [16]. Interestingly, although either F-OVX or F-HFS alone could impair LV contractile function and cardiac autonomic regulation, the acceleration of this impairment was shown in F-HFO rats. This finding was consistent with our previous study that obesity and insulin resistance both accelerate and aggravate the impairment of cardiac contractile function and cardiac sympathovagal balance under estrogen deprivation [36].

### There are clear sex differences in the temporal alteration of cardiometabolic and cardiac autonomic functions

In the present study, we have shown that sex hormone deprivation, obese-insulin resistance or combined condition could impair cardiac contractile function and cardiac sympathovagal balance in both genders. However, there is a clear sexual dimorphism in the temporal alteration of cardiometabolic and cardiac autonomic functions. Although testosterone deprivation did not alter metabolic parameters, it significantly reduced LV contractile function and impaired cardiac autonomic balance as observed as early as week 4. In contrast, there was earlier development of cardiac autonomic imbalance in the F-HFO rats at week 8 suggesting that in estrogen-deprived condition obese-insulin resistance accelerated the development of cardiac autonomic imbalance. We previously demonstrated that cardiac autonomic imbalance was associated with increased sympathetic tone and ROS production [37]. Moreover, increased MDA and ROS levels could impair cardiac autonomic balance via ROS-induced cardiac sympathetic overactivity [38].

Mitochondria are known as the cellular energy factories, and they also participate in many other functions including ROS production. In addition, cardiac mitochondria are crucial to maintain proper cardiac contractile function [39]. Interestingly, a strong tissue and sex specificity was detected in mitochondria. In female cardiomyocytes, mitochondria produce less ROS and have a higher capacity of antioxidant defenses than the male ones [40–42]. Moreover, female cardiac mitochondria have a greater mitochondrial calcium retention capacity compared with males [43]. Therefore, sexual dimorphism of cardiac mitochondria may contribute to differences in the temporal development of cardiometabolic and cardiac autonomic impairments as shown in the present study. Our study limitation was that we did not directly measure the oxidative function of cardiac mitochondria. However, increased production of cardiac mitochondrial ROS level, membrane depolarization and swelling have a direct correlation with oxidative stress-induced mitochondrial dysfunction. Thus, future studies are required to elucidate the impacts of sexual dimorphism on cardiac mitochondrial respiratory function and aerobic capacity under these conditions.

## Conclusions

This study demonstrated that following sex hormone deprivation there is an obvious sex-associated impact on cardiac contractile and mitochondrial functions in the presence of obese-insulin resistance. Specifically, under sex hormone-deprived condition, females are generally more susceptible to develop metabolic disorders, whereas males are more susceptible to develop cardiac autonomic, LV and mitochondrial dysfunction even in the absence of obesity and insulin-resistant condition. However, in the presence of estrogen deprivation condition, the severity of these impairments was further accelerated and aggravated in obese-insulin-resistant females. Our findings suggest that different therapeutic strategies could be targeted for men and women due to sexual dimorphism.
